# Expression of High-Affinity IgE Receptor on Human Peripheral Blood Dendritic Cells in Children

**DOI:** 10.1371/journal.pone.0032556

**Published:** 2012-02-23

**Authors:** Monica Vasudev, Dorothy S. Cheung, Hannah Pincsak, Shun-Hwa Li, Ke Yan, Pippa Simpson, Trivikram Dasu, Mitchell H. Grayson

**Affiliations:** Department of Pediatrics, Medical College of Wisconsin, Milwaukee, Wisconsin, United States of America; University Hospital Freiburg, Germany

## Abstract

**Background:**

In a mouse model of viral induced atopic disease, expression of FcεRI on dendritic cells is critical. While adult human conventional (cDC) and plasmacytoid (pDC) dendritic cells have been shown to express FcεRI, it is not known if this receptor is expressed in childhood and how its expression is governed by IgE.

**Methods:**

Following informed consent of subjects (n = 27, aged 12–188 months), peripheral blood was stained for surface expression of CD19, ILT7, CD1c, IgE, FcεRI and analyzed by flow cytometry (cDC: CD19^−^ ILT7^−^ CD1c^+^; pDC: CD19^−^ ILT7^+^ CD1c^−^). Total and specific serum IgE levels to food and inhalant allergens were determined by ImmunoCAP, and the relationship between FcεRI expression on dendritic cells and sensitization, free IgE, cell bound IgE, and age was determined.

**Results:**

Independent of sensitization status, FcεRI expression was noted on cDC and pDC as early as 12 months of age. Serum IgE level correlated with expression of FcεRI on cDC, but not pDC. Based on the concentration of IgE, a complex relationship was found between surface bound IgE and expression of FcεRI on cDC. pDC exhibited a linear relationship of FcεRI expression and bound IgE that was consistent through all IgE concentrations.

**Conclusions:**

In children, FcεRI expression on cDC and pDC is modulated differently by serum and cell bound IgE. IgE governance of FcεRI expression on cDC depends upon a complex relationship. Further studies are needed to determine the functional roles of FcεRI on cDC and pDC.

## Introduction

Severe viral respiratory infections early in life are associated with increased risk of asthma and atopic disease [1], [2], [3], [4]. Using a mouse model we defined potential mechanisms translating anti-viral immune responses into atopic disease. Mice infected with the murine type 1 parainfluenza virus develop an acute inflammatory response and severe weight loss in the first week of infection. By 10 days post-viral inoculation, the animals clear virus and begin to gain weight; however, by day 21 post-inoculation they manifest chronic airway hyper-reactivity and IL-13 dependent mucous cell metaplasia [5]. In addition, exposure to an environmental antigen during the viral infection is sufficient to generate IgE against the environmental antigen leading to worse atopic disease [6]. We have also shown that a similar response can occur in the gastrointestinal tract [7].

One hallmark of our model is that murine cDC and pDC do not express FcεRI except during severe viral infections, when cognate interactions with a specific neutrophil subset drives FcεRI expression [8]. Cross-linking FcεRI bound IgE on cDC leads to recruitment of IL-13 producing Th2 cells driving the atopic response. We have shown in humans that cross-linking IgE on cDC produces CCL28, the same Th2 chemoattractant critical for the mouse model [9]. Although adult human cDC and pDC are known to express FcεRI, very little is known about induction and regulation of this receptor on human cDC and pDC from children. We undertook this study to answer this issue.

Our study documents expression patterns of FcεRI on peripheral blood cDC and pDC in young children. While the receptors are present early in life, we found a marked differential effect of low and high IgE on cDC FcεRI expression. This finding implies that cDC expressed FcεRI has disparate effects on cDC function depending on serum level of IgE. Further, our data suggest that IgE control of FcεRI may be different in children compared to adults.

## Methods

### Experimental Design

A cross sectional time analysis was employed. Our primary goal was to determine the age at which human cDC and pDC expressed FcεRI. Our secondary goal was to determine if sensitization to any food or inhalant allergen was associated with FcεRI and IgE expression.

### Ethics Statement

This study was approved by the Children's Hospital of Wisconsin IRB. Parental written consent and subject verbal assent was obtained before enrollment of subjects into this study.

### Subjects

Once consent and assent was obtained, an abbreviated atopic history was collected and venipuncture performed. Eligible patients were children aged 1–15 years. Exclusion criteria were: atopic dermatitis, persistent controlled asthma, uncontrolled asthma, immunodeficiency, or any co-morbid disease; use of any corticosteroids within the last month; current therapy with any prescribed medication; use of any investigational agent in the last 30 days; women of childbearing age not on contraception or women breastfeeding.

### Sensitization status and total serum IgE determination

Total IgE and specific IgE levels to foods (casein, alpha-lactalbumin, beta-lactoglobulin, cheese, egg white, cacao, soybean, peanut, codfish, tuna, oat, rice, wheat) and aeroallergens (*D. farinae*, *D. pteronyssinus*, cockroach, cat dander, dog dander, mouse epithelium, rat epithelium, guinea pig epithelium, mouse serum protein, rat serum protein, mouse urine protein, rat urine protein, June grass, *Alternaria tenuis*, *Cladosporium herbarum*, *Aspergillis fumigatus*, common ragweed, oak, birch, *L. destructor*) were determined by ImmunoCAP (Quest Dignostics/IBT Laboratories). Sensitization was defined as elevated serum specific IgE (>0.35 kU/L) to one or more allergens tested.

### Flow Cytometry

**Figure 1:**
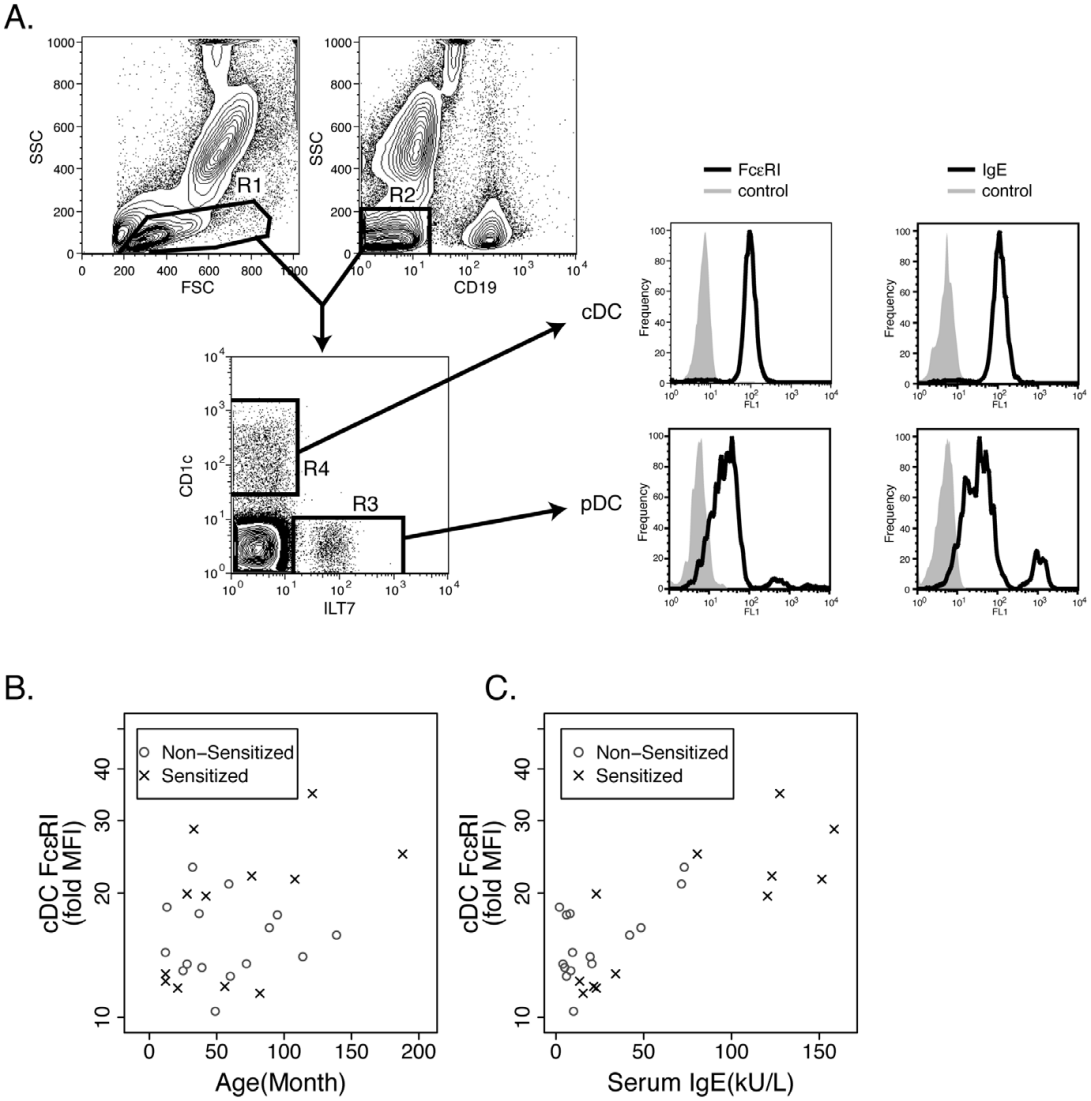
Expression of FcεRI on cDC correlates with serum IgE but not with subject's age. (A) Gating strategy used to identify expression of FcεRI and IgE on cDC and pDC. Cells were gated on scatter (R1) and lack of expression of CD19 (R2, PerCP negative). Those events that satisfied both of these criteria were examined for expression of ILT7 (R3, PE positive) versus CD1c (R4, APC positive). Cells that were CD19^−^ILT7^+^CD1c^−^ were considered pDC and CD19^−^ILT7^−^CD1c^+^ were considered cDC. The expression of FcεRI (left histograms, FITC positive) or IgE (right histograms, FITC positive) on these cells was then determined and compared to an appropriate isotype control. (B) Expression of FcεRI on peripheral blood cDC was determined by flow cytometry and compared to subject's age or (C) serum IgE level. Data are presented as fold MFI FcεRI versus age in months (B) or serum IgE in kU/L (C), with non-sensitized subjects (n = 15) in circles and sensitized subjects (n = 12) in x's. Sensitization was defined as having at least one positive allergen specific IgE by ImmunoCAP.

Immunophenotyping for evaluation of pDC and cDC was performed within 6 hours of specimen collection. 100 µl of heparinized whole blood was stained with antibodies to human CD19 (PerCP labeled clone SJ25C1, BD Biosciences), ILT7 (PE labeled clone 17G10.2, eBioscience), and CD1c (APC labeled clone BDCA-1, Miltenyi Biotec), as well as either antibodies against FcεRIα (FITC labeled clone AER-37, eBioscience) or IgE (FITC labeled, Kirkegaard & Perry Laboratories, Inc.) for 15 min. at room temperature. Erythrocytes were lysed with 2 ml FACSLyse (BD Biosciences) and stained cells were washed with PBS/0.1% NaN_3_ and fixed using 1% paraformaldehyde. Flow cytometry (BD FACSCalibur) was performed with 5×10^5^ to 1×10^6^ total cellular events collected on each stained specimen. cDC (also referred to as mDC-1) were identified as CD19^−^/ILT7^−^/CD1c^+^ and pDC identified as CD19^−^/ILT7^+^/CD1c^−^. This type of phenotyping strategy has been used before, as ILT7 is a specific marker for pDC and CD19^−^CD1c^+^ cells have been shown to be essentially all cDC [9], [10], [11], [12], [13], [14], [15], [16]. Using multi-color flow cytometry and Boolean gating strategy, the expression of IgE and FcεRIα were identified on the cDC and pDC subsets and measured as fold mean florescence (fold MFI) over individual isotype control background using CellQuest Pro software (BD Biosciences) as shown in Figure 1A. Appropriate isotype control antibodies were utilized, and calibration of FcεRIα expression (MEPE) was performed in each run using fluorescent beads as described [17].

### Statistical Analysis

Data were expressed as frequency and percent for categorical variables and median and interquartile range for continuous variables. Since the data were skewed, continuous variables were compared using a Mann-Whitney test while doing 2 group comparisons, and continuous variables were examined using scatter plots and Pearson correlations. To investigate the inter-related effects of variables on the outcomes of interest, a regression tree analysis was done using Salford Systems' CART software (http://salford-systems.com/). Regression trees are a non-parametric approach where the data are split recursively into two groups based on an optimizing function, until the specified limits for subgroups are reached. A least absolute deviance from the median was used as the optimizing function, with a limit of 10 in any group to be split and 5 minimally needed for any group. Linear regression was used to fit the data with linear or a polynomial trend with normality of the error term checked with a normal probability plot. cDC IgE was fit to serum IgE using a non-linear regression for a sigmoidal type curve of the form 1/(1+M/serum IgE)^m^, where M is the (fitted) median value of cDC IgE and m is the slope of the curve at that value. We fit linear regression using SAS 9.2 and non-linear regression using JMP 9.0. Given the relatively small sample, we did not use a bootstrap approach or a holdout sample to validate the model results. Therefore, these results will need to be validated on another sample.

## Results

Twenty-seven patients aged 12 to 188 months were recruited from June through September 2009. All patients denied symptoms of current allergic disease or respiratory tract infection. A summary of subject characteristics is in Table 1. Subjects were classified as sensitized based on ImmunoCap testing, with a single positive test being considered “sensitized”. No correlation was noted between sensitization status and age, and other than total serum IgE there was no significant difference in any demographic information between the sensitized (n = 12) and non-sensitized subjects (n = 15).

**Table 1:** Subject Characteristics.

Variable	Non-sensitized (n = 15)	Sensitized† (n = 12)
Age (months), median (range)	49 (12–139)	49 (12–188)
Male, n (%)	8 (53%)	5 (42%)
Personal History Atopy*, n(%)	14 (93%)	12 (100%)
Family History Atopy*, n(%)	12 (80%)	7 (58%)
Elevated Food specific IgE, n(%)	0 (0%)	9 (75%)§
Elevated Inhalant allergen specific IgE, n(%)	0 (0%)	9 (75%)

*Atopy history: defined as self reported or MD diagnosed allergic rhinitis/hayfever/environmental allergies, asthma/wheeze, atopic dermatitis, food allergy. Family history of atopy was based on self-reporting only.

† Sensitized: defined as any elevated food or inhalant serum specific IgE.

§ Only 4 of these subjects had a clinical history suggestive of an allergy for the foods to which they had IgE against.

In a linear regression, we found that there was a moderate correlation between age and serum IgE (r = 0.53), with about 25% of the variation of IgE explained by the linear relationship with age. However, much of this relationship occurred in the non-sensitized group (r = 0.56) compared to the sensitized group (r = 0.29). This is not surprising, since serum IgE levels can fluctuate, such as with viral infections, and our data were obtained at only a single time point per subject [18].

Comparing the relative frequencies of conventional (cDC) and plasmacytoid dendritic cells (pDC) amongst all of our subjects, we found 1.26 (0.66–2.55) (median (IQR)) fold more cDC than pDC. This ratio was not significantly different between sensitized and non-sensitized subjects, nor did it correlate with age (data not shown). While this ratio of cDC to pDC is lower than what has been reported in older children, it is similar to what has been reported in adult studies using a dendritic cell phenotyping strategy similar to ours [14], [15], [19].

### Conventional dendritic cells (cDC)

We initially hypothesized that similar to the rodent system, peripheral blood cDC would not express FcεRI early in life. However, as shown in Figure 1B, FcεRI expression was detected on cDC at all ages. Given that viral infections have been shown to upregulate expression of FcεRI on dendritic cells, and that older individuals likely had more viral infections, we supposed that expression of the receptor would increase with age [18]. As shown in Figure 1B, the age of the subject had a marginally linear correlation to level of expression of FcεRI on cDC (r = 0.36, p = 0.07) and this marginal correlation was found in sensitized (r = 0.53, p = 0.08) but not non-sensitized (r = −0.002, p = 0.99) subjects. Further, no association was found between family atopic status and FcεRI expression on cDC (p = 0.42, data not shown).

In human mast cells and basophils, IgE modulates FcεRI expression [20]. In fact, in a study with anti-IgE the reduction in IgE directly correlated with subsequent reduction of FcεRI expression on peripheral blood DC of adults [21]. We examined the relationship between serum IgE and cDC expression of FcεRI. As shown in Figure 1C, there was a linear correlation (r = 0.78, p<0.0001) between serum IgE levels and cDC expression of FcεRI. This correlation held regardless of sensitization status (r = 0.83, p = 0.0009 sensitized subjects; r = 0.64 non-sensitized subjects, p = 0.01).

Sensitization status did not differentiate level of expression of FcεRI on cDC (Table 2). However, a regression tree analysis revealed that when serum IgE was less than 60 kU/L, FcεRI expression was significantly higher on cDC from non-sensitized subjects (median (IQR) = 14.03 (13.2, 16.48) fold MFI) compared to sensitized individuals (median (IQR) = 12.05 (11.77, 12.74) fold MFI), p = 0.04.

**Table 2:** Comparison of Sensitized and Non-Sensitized Children for Dendritic Cell Expression of IgE and FcεRI.

	Median (IQR)		
Variable	Non-Sensitized (n = 15)	Sensitized (n = 12)	p-value†
Age (month)	49 (28, 89)	49 (24.5, 95)	>0.99
Serum IgE (kU/L)	9.5 (6, 42)	57.25 (22.3, 125)	0.003
pDC bound IgE (fold MFI)	2.42 (1.54, 2.74)	2.13 (1.15, 4.1)	>0.999
pDC FcεRI (fold MFI)	2.65 (1.59, 3.25)	2.02 (1.04, 3.25)	0.39
cDC bound IgE (fold MFI)	4.3 (2.68. 19.2)	17.33 (5.12, 33.5)	0.08
cDC FcεRI (fold MFI)	14.37 (13.2, 17.8)	19.8 (12.1, 23.5)	0.48

† : Mann-Whitney test.

IQR: interquartile range, cDC: conventional dendritic cells, pDC: plasmacytoid dendritic cells.

**Figure 2:**
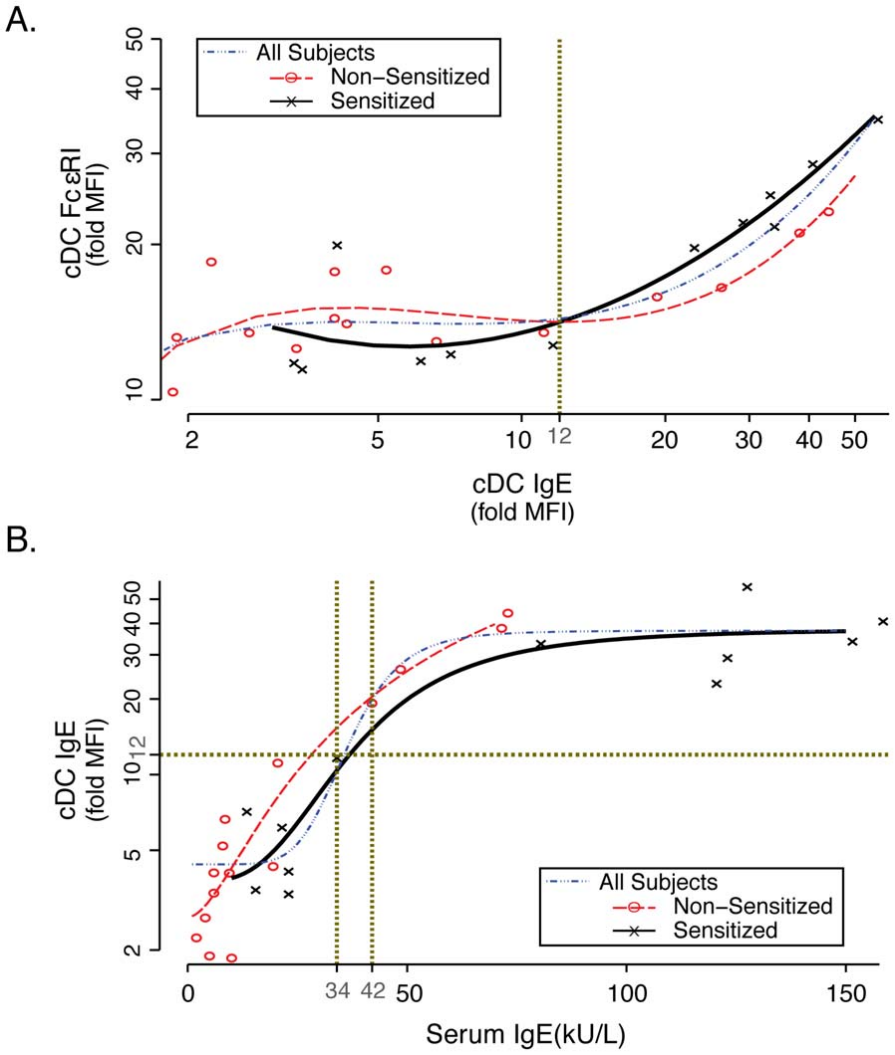
Correlation of cDC expression of FcεRI with IgE. Expression of FcεRI and IgE on the surface of cDC was determined by flow cytometry. (A) Data are presented as fold MFI FcεRI versus fold MFI IgE expression, with non-sensitized subjects (n = 15) in red circles and sensitized subjects (n = 12) in black x's. Note the difference in the slope of the fitted lines (non-sensitized subjects in red dotted line; sensitized subjects in black solid line) above and below a fold MFI IgE of 12, suggesting that there is differential regulation of FcεRI on cDC around this level of cell bound IgE. For all subjects the data was fit with the following cubic equation (blue line): Ln(cDC FcεRI) = 2.26+0.72*ln(cDC IgE)-0.43*ln(cDC IgE)^2^+0.082*ln(cDC IgE)^3^); r = 0.86; p = 0.045. For equations based on sensitization status, see Table 3. (B) Expression of cell bound IgE on cDC compared with serum (free) IgE levels. Data are presented as in (A) with fold MFI IgE expression versus serum IgE (kU/L). Note that a cell bound IgE fold MFI of 12 correlates with a serum IgE of around 38 (based on statistical fitting of the curve, this value could range between 34 and 42 kU/L).

Since bound IgE modulates expression of FcεRI on human mast cells and basophils, we examined the level of IgE on peripheral blood cDC and correlated this with FcεRI expression. As shown in Figure 2A, the relationship between cDC expression of FcεRI and surface bound IgE was complex. With low level staining of IgE (less than a fold MFI IgE expression of 12) there was no relationship between surface bound IgE and FcεRI. However, at higher levels of surface bound IgE there was a direct correlation between bound IgE and FcεRI expression. Interestingly, fitting curves to the data (the equations are shown in Table 3) showed the relationship between cDC bound IgE and FcεRI expression to be different for sensitized and non-sensitized individuals. In Figure 2A the relationship in sensitized individuals appears to be exponential, while in non-sensitized it was more sigmoidal. However, both curves appear to converge when subjects expressed a fold MFI for IgE of 12 or greater. Analyzing all of the data together (without making a distinction on sensitization status) generated a curve that was sigmoidal and the inflection point remained at a fold MFI IgE expression of 12 (Figure 2A, blue line).

**Table 3:** Sensitization status regression models with significant Pearson correlations.

	Regression Equation	Pearson correlation (p-value)
**Non-sensitized**		
pDC FcεRI Vs. pDC IgE		
	Ln(pDC FcεRI) = 0.26+0.73*ln(pDC IgE)	r = 0.76 (p = 0.001)
cDC FcεRI Vs. cDC IgE		
	Ln(cDC FcεRI) = 2.49+0.13*ln(cDC IgE)	r = 0.65 (p = 0.009)
**Sensitized**		
pDC FcεRI Vs. pDC IgE		
	Ln(pDC FcεRI) = 0.004+0.8*ln(pDC IgE)	r = 0.98 (p<0.0001)
cDC FcεRI Vs. cDC IgE		
	Ln(cDC FcεRI) = 2.06+0.32*ln(cDC IgE)	r = 0.85 (p = 0.0004)

Ln indicates the natural log transformation.

By overlaying serum IgE levels, we found only subjects with a serum IgE above 34–42 kU/L had a cDC fold IgE expression of 12 or greater (Figure 2B). These data suggest serum IgE drives FcεRI expression on peripheral blood cDC only when it crosses this threshold level. Because the two curves converge (Figure 2A), this effect appears to be unrelated to subject's sensitization status.

### Plasmacytoid dendritic cells (pDC)

**Figure 3:**
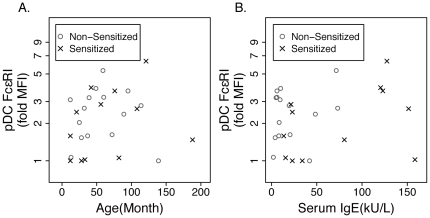
Expression of FcεRI on pDC does not correlate with serum IgE or age of subject. Expression of FcεRI on peripheral blood pDC was determined by flow cytometry and compared to subject's age (panel A) and serum IgE level (panel B). Data are presented as in Figure 1.

Unlike peripheral blood cDC, pDC expression of FcεRI was minimal (median (IQR) = 15.82 (12.74–21.05) versus 2.47 (1.47–3.25) on cDC and pDC, respectively). In fact, 6 of the 27 subjects appeared to have no expression of FcεRI on their pDC (fold MFI equal to or less than 1.0). As shown in Table 2, analogous to the cDC data, sensitization status did not associate with level of expression of FcεRI on pDC. We also found no linear correlation between age of the subject and pDC expression of FcεRI (Figure 3A; r = 0.13, p = 0.51 for all subjects; r = 0.28, p = 0.38 for sensitized subjects; and r = −0.05, p = 0.87 for non-sensitized subjects). In sum, there was no correlation between a given subject's expression of FcεRI on their cDC or pDC and age.

Unlike with cDC (Figure 1C), serum IgE levels did not correlate with pDC expression of FcεRI (Figure 3B, r = 0.21, p = 0.29 for all subjects; r = 0.42, p = 0.17 for sensitized subjects; r = 0.21, p = 0.46 for non-sensitized subjects). Also, unlike cDC, pDC bound IgE levels correlated linearly with pDC expression of FcεRI in both sensitized (r = 0.76, p = 0.001) and non-sensitized subjects (r = 0.98, p<0.0001). These data provide further evidence for a direct association between bound IgE and FcεRI expression in pDC but not cDC, suggesting that regulation of FcεRI is dissimilar in cDC and pDC.

## Discussion

Other studies have attempted to describe relationships between serum IgE, cell-bound IgE, and FcεRI expression on dendritic cells [17], [21]. These prior studies utilized adults, and did not examine expression in children. There is one publication in the literature that looked at children [22]. However, this report examined older children and did not differentiate FcεRI and IgE staining on cDC and pDC, as they used CD11c and MHC-II as markers for dendritic cells—markers that are insufficient to distinguish cDC and pDC cells in humans [23]. Further, our study utilized regression analysis to better understand the role of FcεRI on dendritic cells in children of various ages. We found that as early as 12 months of age, regardless of sensitization status, FcεRI is expressed on peripheral blood cDC and pDC, although pDC expression of some subjects was quite minimal. No association between age and expression of IgE or FcεRI was found, indicating that our initial hypothesis was wrong (i.e., FcεRI expression on dendritic cells would correlate with age). Although we do acknowledge that a longitudinal study would be more appropriate to examine the relationship between age and receptor expression. It does remain possible that early life developmental control of FcεRI expression exists before 12 months of age—especially with pDC. Once induced, FcεRI expression clearly can be modulated by viral infections [18].

Interestingly, we found no association between sensitization status and FcεRI expression. We cannot equate sensitization with atopy because we did not challenge our subjects with the respective allergens. Further, it is quite possible that those subjects we identified as non-sensitized were indeed making specific IgE—just against antigens that were not included in our panels. For these reasons our data may be different from two studies in adults where atopic subjects were noted to have significantly higher FcεRI expression on cDC and pDC [15], [21]. Our results do corroborate those of the adult studies in the fact that expression of FcεRI is much higher on cDC than on pDC. What the functional implications are for this difference in expression remains unclear.

The most intriguing finding of our study is the major difference in the role of IgE in receptor expression. Whereas a direct relationship between FcεRI expression and IgE on both cDC and pDC have been reported by others, we found that only pDC FcεRI expression correlated in a linear fashion with bound IgE [21], [24]. Our data did not show a relationship between serum IgE and FcεRI expression on pDC. This suggests free IgE may not drive receptor expression, an event that depends singularly upon level of bound IgE. This difference may be due to the low level of expression of FcεRI on pDC, and may explain differences between how IgE drives FcεRI expression on mast cells and basophils when compared to pDC [20], [25].

Unlike pDC, expression of FcεRI on cDC correlated directly with serum IgE levels. However, the relationship between bound IgE and FcεRI was more complex. When cell bound IgE was below 12 fold MFI there was little effect of bound IgE on FcεRI expression. Once bound IgE levels crossed the threshold, a direct relationship developed. At low levels of serum IgE (<34–42 kU/L) there is rapid accumulation of bound IgE with little effect on FcεRI expression. At these low levels of IgE, a difference in correlation of bound IgE and FcεRI exists between sensitized and non-sensitized subjects. Both responses are parabolic, however, non-sensitized subjects tend to have an initial increase in FcεRI expression with increasing bound IgE (below 12 fold MFI). Sensitized subjects had the exact opposite effect. Once serum IgE levels reached 34–42 kU/L, corresponding to bound IgE levels of 12 fold MFI, both sensitized and non-sensitized subjects exhibited a direct correlation between IgE and FcεRI.

We have found a threshold effect of bound IgE on cDC FcεRI. The functional implications for this threshold effect are unclear; it is inviting to speculate that FcεRI has disparate functions at low versus high IgE levels. Perhaps at lower levels FcεRI acts as a rheostat, similar to what is seen in rodents, where the receptor functions as a mechanism for cellular recruitment and less for antigen-uptake [5], [6]. At higher levels of IgE, the receptor may function more as a means for “antigen-focusing” [26]. It is beyond the scope of this study to elucidate actual mechanisms behind this phenomenon, but the functional implications of IgE regulation of FcεRI expression are tantalizing.

Our study had several limitations. First and foremost, we utilized statistical modeling to evaluate our data, and, as a result, it is important that these findings be validated in a second cohort. Further, our sample size was relatively small and likely limited our ability to detect subtle differences. We did not enroll infants younger than 12 months of age, and we were unable to determine if viral infection relates to induction of FcεRI. Finally, defining sensitization status by selected ImmunoCAP may not be ideal—especially from a clinical perspective. However, given that our results were mostly independent of sensitization status, we believe this is not a major defect. Indeed, most of the limitations of our study would only lessen our ability to define statistically significant differences. Since we were able to document clear relationships between IgE (serum and cell bound) and cDC and pDC expression of FcεRI despite these limitations, their presence only strengthen the importance of our findings.

In summary, our results demonstrate the presence of FcεRI on peripheral blood cDC and pDC in children as young as 1 year old, which is in contrast to what is seen in the rodent system. Our data also provide evidence of differential regulation of FcεRI on cDC and pDC in children. With cDC a complex inter-relationship between serum IgE, cell bound IgE, and expression of FcεRI exists that appears to hinge on an IgE level of 34–42 kU/L. The functional significance of this relationship is unclear, and we do not yet understand the mechanisms responsible for this relationship. Future studies need to be undertaken to examine expression of FcεRI on cDC and how IgE modulates this receptor and cDC function.

## References

[1] Sigurs N, Aljassim F, Kjellman B, Robinson PD, Sigurbergsson F (2010). Asthma and allergy patterns over 18 years after severe RSV bronchiolitis in the first year of life.. Thorax.

[2] Sigurs N, Gustafsson PM, Bjarnason R, Lundberg F, Schmidt S (2005). Severe respiratory syncytial virus bronchiolitis in infancy and asthma and allergy at age 13.. Am J Respir Crit Care Med.

[3] Escobar GJ, Ragins A, Li SX, Prager L, Masaquel AS (2010). Recurrent wheezing in the third year of life among children born at 32 weeks' gestation or later: relationship to laboratory-confirmed, medically attended infection with respiratory syncytial virus during the first year of life.. Arch Pediatr Adolesc Med.

[4] Johnston SL, Pattemore PK, Sanderson G, Smith S, Campbell MJ (1996). The relationship between upper respiratory infections and hospital admissions for asthma: a time-trend analysis.. Am J Respir Crit Care Med.

[5] Grayson MH, Cheung D, Rohlfing MM, Kitchens R, Spiegel DE (2007). Induction of high-affinity IgE receptor on lung dendritic cells during viral infection leads to mucous cell metaplasia.. J Exp Med.

[6] Cheung DS, Ehlenbach SJ, Kitchens T, Riley DA, Grayson MH (2010). Development of atopy by severe paramyxoviral infection in a mouse model.. Ann Allergy Asthma Immunol.

[7] Chen X, Leach D, Hunter DA, Sanfelippo D, Buell EJ (2011). Characterization of intestinal dendritic cells in murine norovirus infection.. Open Immunol J.

[8] Cheung DS, Ehlenbach SJ, Kitchens RT, Riley DA, Thomas LL (2010). Cutting edge: CD49d+ neutrophils induce FcepsilonRI expression on lung dendritic cells in a mouse model of postviral asthma.. J Immunol.

[9] Khan SH, Grayson MH (2010). Cross-linking IgE augments human conventional dendritic cell production of CC chemokine ligand 28.. J Allergy Clin Immunol.

[10] Rissoan MC, Duhen T, Bridon JM, Bendriss-Vermare N, Peronne C (2002). Subtractive hybridization reveals the expression of immunoglobulin-like transcript 7, Eph-B1, granzyme B, and 3 novel transcripts in human plasmacytoid dendritic cells.. Blood.

[11] Cao W, Bover L (2010). Signaling and ligand interaction of ILT7: receptor-mediated regulatory mechanisms for plasmacytoid dendritic cells.. Immunol Rev.

[12] Cao W, Bover L, Cho M, Wen X, Hanabuchi S (2009). Regulation of TLR7/9 responses in plasmacytoid dendritic cells by BST2 and ILT7 receptor interaction.. J Exp Med.

[13] Cao W, Rosen DB, Ito T, Bover L, Bao M (2006). Plasmacytoid dendritic cell-specific receptor ILT7-Fc epsilonRI gamma inhibits Toll-like receptor-induced interferon production.. J Exp Med.

[14] Kirsche H, Niederfuhr A, Deutschle T, Fuchs C, Riechelmann H (2010). Ratio of myeloid and plasmacytoid dendritic cells and TH2 skew in CRS with nasal polyps.. Allergy.

[15] Lundberg K, Greiff L, Borrebaeck CA, Lindstedt M (2010). FcepsilonRI levels and frequencies of peripheral blood dendritic cell populations in allergic rhinitis.. Hum Immunol.

[16] Patterson S, Donaghy H, Amjadi P, Gazzard B, Gotch F (2005). Human BDCA-1-positive blood dendritic cells differentiate into phenotypically distinct immature and mature populations in the absence of exogenous maturational stimuli: differentiation failure in HIV infection.. J Immunol.

[17] Foster B, Metcalfe DD, Prussin C (2003). Human dendritic cell 1 and dendritic cell 2 subsets express FcepsilonRI: correlation with serum IgE and allergic asthma.. J Allergy Clin Immunol.

[18] Subrata LS, Bizzintino J, Mamessier E, Bosco A, McKenna KL (2009). Interactions between innate antiviral and atopic immunoinflammatory pathways precipitate and sustain asthma exacerbations in children.. J Immunol.

[19] Silver E, Yin-DeClue H, Schechtman KB, Grayson MH, Bacharier LB (2009). Lower levels of plasmacytoid dendritic cells in peripheral blood are associated with a diagnosis of asthma 6 yr after severe respiratory syncytial virus bronchiolitis.. Pediatr Allergy Immunol.

[20] Saini S, MacGlashan DW, Adelman D, Jardieu P, Togias A (1997). Culture with IgE increases IgE density and FcεRI expression on human basophils.. J Allergy Clin Immunol.

[21] Prussin C, Griffith DT, Boesel KM, Lin H, Foster B (2003). Omalizumab treatment downregulates dendritic cell FcepsilonRI expression.. J Allergy Clin Immunol.

[22] Dehlink E, Baker AH, Yen E, Nurko S, Fiebiger E (2010). Relationships between levels of serum IgE, cell-bound IgE, and IgE-receptors on peripheral blood cells in a pediatric population.. PLoS One.

[23] Grayson MH (2006). Lung dendritic cells and the inflammatory response.. Ann Allergy Asthma Immunol.

[24] Schroeder JT, Bieneman AP, Chichester KL, Hamilton RG, Xiao H (2010). Decreases in human dendritic cell-dependent T(H)2-like responses after acute in vivo IgE neutralization.. J Allergy Clin Immunol.

[25] Thompson HL, Metcalfe DD, Kinet JP (1990). Early expression of high-affinity receptor for immunoglobulin E (Fc epsilon RI) during differentiation of mouse mast cells and human basophils.. J Clin Invest.

[26] Jurgens M, Wollenberg A, Hanau D, de la Salle H, Bieber T (1995). Activation of human epidermal Langerhans cells by engagement of the high affinity receptor for IgE, Fc epsilon RI.. J Immunol.

